# Diversity of Betaproteobacteria revealed by novel primers suggests their role in arsenic cycling

**DOI:** 10.1016/j.heliyon.2019.e03089

**Published:** 2020-01-02

**Authors:** Anirban Chakraborty, Chanchal K. DasGupta, Punyasloke Bhadury

**Affiliations:** a Department of Life Science and Biotechnology, Jadavpur University, Kolkata, 700032, West Bengal, India; b Integrative Taxonomy and Microbial Ecology Research Group, Department of Biological Sciences and Centre for Climate and Environmental Studies, Indian Institute of Science Education and Research Kolkata, Mohanpur, Nadia, 741246, West Bengal, India

**Keywords:** Environmental science, Environmental geochemistry, Hydrology, Groundwater, Microbiology, Bacteria, Microorganism, Primers, 16S rRNA gene, Betaproteobacteria, Aquifer, Arsenic

## Abstract

High arsenic concentration in groundwater is a severe environmental problem affecting human health, particularly in countries of South and South-East Asia. The Bengal Delta Plain (BDP) distributed within India and Bangladesh is a major arsenic-affected region where groundwater is the primary source of drinking water. Previous studies have indicated that members of the bacterial class *Betaproteobacteria* constitute a major fraction of the microbial community in many of the aquifers within this region. Bacteria belonging to this class are known to be involved in redox cycling of arsenic as well as other metals such iron and manganese, thereby impacting arsenic mobilization and immobilization. While microbial diversity in arsenic-contaminated environments is generally assessed using universal 16S rRNA gene primers, targeted evaluation of Betaproteobacteria diversity remains poorly constrained. In this study, bacterial diversity was investigated in the groundwater from two shallow aquifers (West Bengal, India) based on 16S rRNA gene clone libraries and sequencing using a custom-designed pair of primers specific to *Betaproteobacteria*. Specificity of the primers was confirmed *in silico* as well as by the absence of PCR amplification of other bacterial classes. Four major families (*Burkholderiaceae*, *Comamonadaceae*, *Gallionellaceae* and *Rhodocyclaceae*) were detected among which members of *Burkholderiaceae* represented 59% and 71% of the total community in each aquifer. The four OTUs (operational taxonomic units; 97% sequence identity) within *Burkholderiaceae* were close phylogenetic relatives of bacteria within the genus *Burkholderia* known to solubilize phosphate minerals. Additionally, the OTUs belonging to *Gallionellaceae* were closely related to the members of the genera *Gallionella* and *Sideroxydans*, known to oxidize iron under microaerophilic conditions. These results suggest that members of *Betaproteobacteria* can potentially influence iron and phosphorus cycling which can influence biogeochemistry in arsenic-contaminated aquifers of the BDP.

## 1 Introduction

Elevated natural concentrations of arsenic (As) in groundwater is a major environmental health problem in many South and South-East Asian countries including India and Bangladesh, where consumption of As-contaminated water in the fertile Bengal Delta Plain (BDP) affects millions of people on a daily basis [1, 2]. In particular, the extent of contamination is greater in the shallow Holocene grey sand aquifers which supply majority of the drinking water in these regions, whereas groundwater in the deeper Pleistocene brown sand aquifers is largely known to be arsenic-free [3]. The primary source of arsenic in the BDP aquifers is considered to be geogenic, the most widely accepted explanation being the release of adsorbed and co-precipitated As from iron (Fe) and manganese (Mn) oxyhydroxides by microbially mediated reductive dissolution [4]. On the other hand, arsenic can be microbially immobilized by arsenic- and iron-oxidizing bacteria through direct enzymatic oxidation [5] or adsorption onto biogenic iron oxides [6]. A number of environmental parameters such as biodegradable organic matter [7], iron [8], phosphate [9] and other electron acceptors [10, 11] have been shown to influence microbial arsenic cycling as well as groundwater microbial community composition and functions [12]. Therefore, an in-depth understanding of the composition and distribution of indigenous microbial communities in BDP aquifers is crucial, particularly in the context of developing cost-effective long-term arsenic mitigation strategies and for supply of arsenic free potable water.

Over the years, several investigations of microbial communities in the BDP aquifers have shown diversity in microbial assemblages spatially (across aquifers) as well as temporally (e.g. seasonal influences such as precipitation) within the same broad geographic location [13, 14, 15, 16, 17]. In 2009, an investigation of microbial communities in shallow and deep tube wells in Bangladesh revealed that bacteria belonging to the class *Betaproteobacteria* were clearly the dominant members, constituting up to ~84% of the entire community [13]. Another study by Sultana *et al* showed greater abundance of *Betaproteobacteria* in a shallow (21m) monitoring well compared to that in a deeper (85m) well [14]. Similar observations were reported by Hassan *et al* where abundance of *Betaproteobacteria* was observed as the most abundant class (~69% of the community) in a comparative examination of microbial communities from 24 groundwater samples in Bangladesh [17]. In addition to BDP aquifers, this bacterial class also has been shown to dominate the microbial communities in As-contaminated soils from China and the United Kingdom [18]. Taken together, these observations are indicative of an emerging pattern where members of *Betaproteobacteria* are major inhabitants of arsenic-contaminated subsurface in geographically distant regions. Comprising over 75 genera and 400 species, *Betaproteobacteria* is a large class within the phylum *Proteobacteria* [19]. In addition, members of *Betaproteobacteria* are frequently detected in subsurface environments in general and display an enormous range of metabolic diversity such as redox transformation of As [5] and Fe [20], denitrification [21] and biodegradation of recalcitrant organic compounds [22]. Investigations focusing on *Betaproteobacteria* diversity are therefore critical to better understand the potential of members of this class in arsenic cycling within contaminated aquifers.

In India, arsenic contamination in the BDP extends into the state of West Bengal, where the Nadia district is one of the severely impacted regions. The shallow grey sand aquifers in this region contain much higher As concentrations than the deeper brown sand aquifers [23]. Previous geochemical data has shown marked heterogeneity in the distribution of dissolved organic carbon in these aquifers, with greater input and preferential biodegradation of mature hydrocarbons as well as increased Fe and Mn concentrations in the shallow aquifers [24]. A recent investigation of bacterial community structures used clone libraries of the 16S rRNA gene and the arsenic oxidase large subunit *aioA* gene to demonstrate that *Betaproteobacteria* continued to dominate microbial communities in two shallow aquifers in the Nadia district [16]. A number of genera such as *Hydrogenophaga* and *Albidiferax* were prominent in the *aioA* clone library, which strongly suggested involvement of these microorganisms in As oxidation. On the other hand, the 16S rRNA gene clone library indicated presence of genera like *Acidovorax* and *Dechloromonas*, which are known to oxidize Fe(II) under anoxic conditions [25]. Interestingly, the above genera have also been detected in hydrocarbon-contaminated aquifers [26]. Relative abundance of the above bacterial groups was observed to be seasonally variable, likely due to monsoon-induced changes in groundwater.

The objective of the current study was to develop a community fingerprinting tool specific towards the class *Betaproteobacteria*. Taxon-specific primers have been shown to improve detection capability of less abundant organisms in targeted diversity analyses using community fingerprinting methods [27]. In the current study, a pair of robust degenerate *Betaproteobacteria*-specific 16S rRNA gene primers was custom designed. Additionally, groundwater from the two shallow aquifers described above was re-sampled and clone library analyses were conducted using the above new pair of primers. These analyses showed that the *Betaproteobacteria* communities in both wells were massively dominated by members of the family *Burkholderiaceae*, which was not previously detected in these wells. Additionally, the new primers were able to detect the presence of *Sideroxydans*-like organisms that are well known for microaerophilic Fe oxidation. These primers therefore can provide deeper understanding of the functioning of members of *Betaproteobacteria* in arsenic contaminated aquifers.

## 2 Materials and methods

### 2.1 Groundwater sampling and geochemical analyses

**Figure 1:**
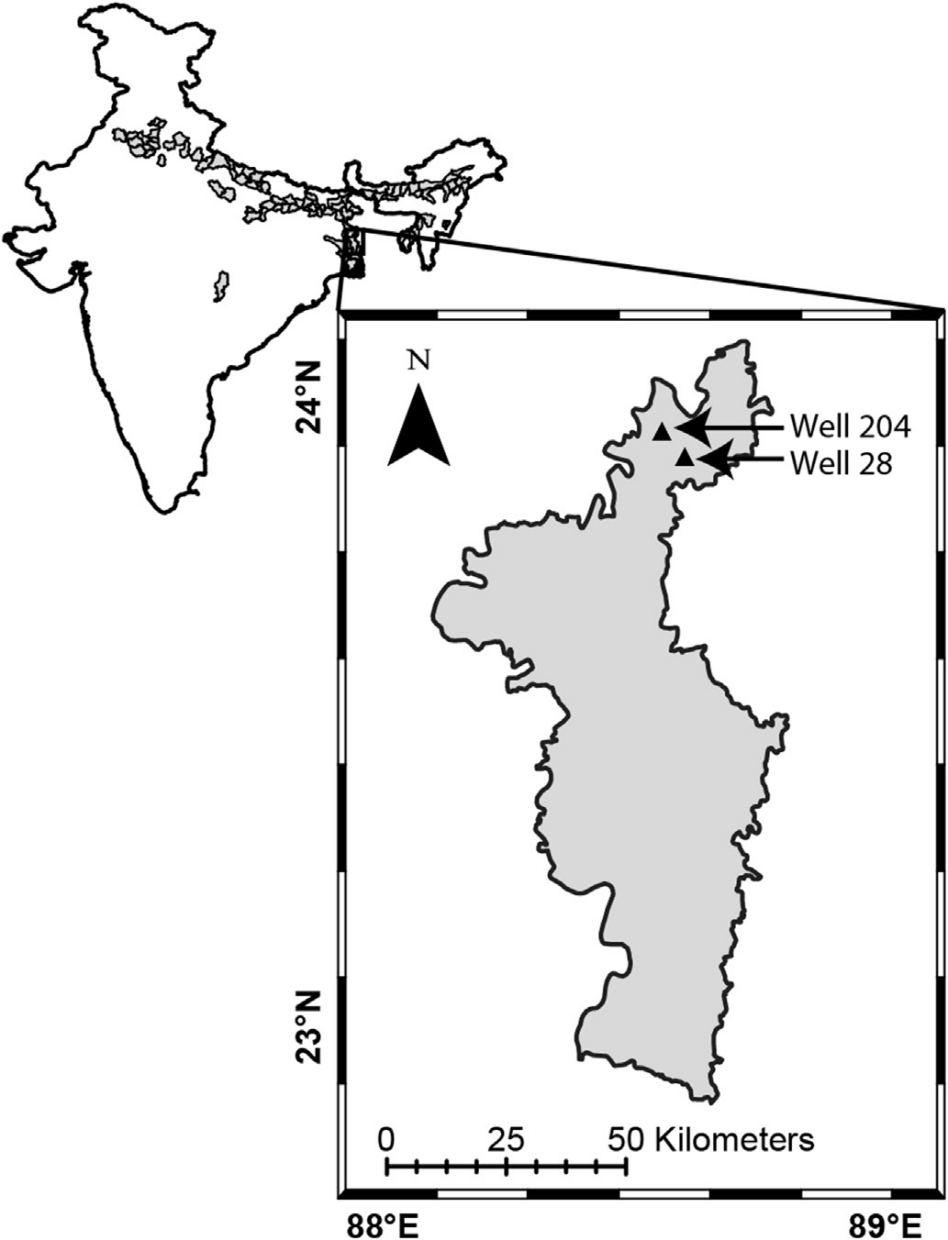
Map of mainland India highlighting the districts where groundwater is As-affected along with a blowup of Nadia district, showing the locations of the sampling wells in the Karimpur II block.

In November 2013, groundwater was collected from two As-rich grey sand shallow aquifers (Well 28 and Well 204) located in Karimpur Block II of Nadia district in the state of West Bengal, India (Figure 1). A detailed geological description of these aquifers and groundwater sampling methods has been provided elsewhere [16]. Briefly, fresh groundwater was collected from these two wells after pumping out roughly three times the well volumes including the stagnant water in the wells. Groundwater temperature, pH and total dissolved solids (TDS) were measured immediately after collection. In addition, two sets of water samples (50 mL) were sterile filtered (syringe filter, 0.45μm pore size) and stored in sterile falcon tubes for further geochemical analyses of major ions and metals. Colorimetric assays were subsequently used in the laboratory to measure dissolved nitrate [28], dissolved phosphate [29] as well as total Fe [30] and As [31].

Two liters of groundwater from each well were preserved with absolute molecular grade ethanol (2% final concentration) immediately after collection to limit microbial activity and prevent denaturation of nucleic acids. The samples were immediately transported to laboratory and passed through 0.22 μm pore size Sterivex filters (Millipore Sigma, Danvars, MA, USA) using a peristaltic pump for concentrating the biomass. The filters were stored at -20 °C and subjected to environmental DNA extraction following a protocol described elsewhere [32].

### 2.2 Designing 16S rRNA gene primers specific to class *Betaproteobacteria*

Twenty seven near-full-length 16S rRNA gene sequences representing all known families of the class *Betaproteobacteria* were downloaded from nucleotide databases (GenBank/ENA/DDBJ), along with 9 additional sequences representative of other classes in the phylum *Proteobacteria* (Table 1). These 36 sequences were subsequently aligned in ClustalW [33] for identification of regions conserved only within the class *Betaproteobacteria*. Based on this alignment, a new set of forward and reverse primers Beta52F (5′ AAGTCGAACGGYARCRSRG 3′) and Beta1014R (5′ GTGCYCGAAAGRGARCYK 3’), respectively, were designed. The primers were subsequently examined using probeCheck web server [34] for self-complementation, low GC content and low Tm value to ensure sufficient thermal window for efficient annealing. The size of 16S rRNA gene amplicons generated using these primers were approximately 950bp.

**Table 1:** The names, NCBI Accession Numbers and taxonomy of the 36 bacterial species (27 belonging to *Betaproteobacteria*) which were chosen for designing the pair of primers used in this study.

	Accession Number#	Domain	Phylum	Class	Order	Family	Genus
Organism (Betaproteobacteria)							
*Acidovorax delafieldii* strain 2AN	HM625980	Bacteria	Proteobacteria	Betaproteobacteria	Burkholderiales	Comamonadaceae	*Acidovorax*
*Acidovorax ebreus* strain TPSY	CP001392	Bacteria	Proteobacteria	Betaproteobacteria	Burkholderiales	Comamonadaceae	*Acidovorax*
*Dechloromonas agitata* strain CKB	NR_024884	Bacteria	Proteobacteria	Betaproteobacteria	Rhodocyclales	Rhodocyclaceae	*Dechloromonas*
*Dechloromonas aromatica* strain RCB	NC_007298	Bacteria	Proteobacteria	Betaproteobacteria	Rhodocyclales	Rhodocyclaceae	*Dechloromonas*
*Azospira suillum* strain PS	AF170348	Bacteria	Proteobacteria	Betaproteobacteria	Rhodocyclales	Rhodocyclaceae	*Azospira*
*Sideroxydans lithotrophicus* strain ES-1	NR_074731	Bacteria	Proteobacteria	Betaproteobacteria	Nitrosomonadales	Gallionellaceae	*Sideroxydans*
*Sideroxydans lithotrophicus* strain LD-1	DQ386859	Bacteria	Proteobacteria	Betaproteobacteria	Nitrosomonadales	Gallionellaceae	*Sideroxydans*
*Leptothrix discophora* strain SS-1	NR_025916	Bacteria	Proteobacteria	Betaproteobacteria	Burkholderiales	Comamonadaceae	*Leptothrix*
*Ferritrophicum radicicola* strain CCJ	DQ386263	Bacteria	Proteobacteria	Betaproteobacteria	Ferritrophicales	Ferritrophicaceae	*Ferritrophicum*
*Pseudogulbenkiania* sp. strain 2002	AY609199	Bacteria	Proteobacteria	Betaproteobacteria	Neisseriales	Chromobacteriaceae	*Pseudogulbenkiania*
*Comamonas denitrificans* isolate SG15	HE681736	Bacteria	Proteobacteria	Betaproteobacteria	Burkholderiales	Comamonadaceae	*Comamonas*
*Chromobacterium violaceum* strain ATCC 12472	NC_005085	Bacteria	Proteobacteria	Betaproteobacteria	Neisseriales	Chromobacteriaceae	*Chromobacterium*
*Ralstonia pickettii* strain HM-1	DQ997838	Bacteria	Proteobacteria	Betaproteobacteria	Burkholderiales	Burkholderiaceae	*Ralstonia*
*Rhodoferax fermentans* strain FR2	NR_025840	Bacteria	Proteobacteria	Betaproteobacteria	Burkholderiales	Comamonadaceae	*Rhodoferax*
*Aquaspirillum serpens* strain IAM 13944	NR_040895	Bacteria	Proteobacteria	Betaproteobacteria	Neisseriales	Chromobacteriaceae	*Aquaspirillum*
*Alcaligenes* sp. strain X9-3	KC156906	Bacteria	Proteobacteria	Betaproteobacteria	Burkholderiales	Alcaligenaceae	*Alcaligenes*
*Sulfuricella denitrificans* strain skB6	AB506456	Bacteria	Proteobacteria	Betaproteobacteria	Sulfuricellales	Sulfuricellaceae	*Sulfuricella*
*Thiobacillus denitrificans* strain NCIMB 9548	NR_025358	Bacteria	Proteobacteria	Betaproteobacteria	Hydrogenophilales	Hydrogenophilaceae	*Thiobacillus*
*Methylophilus methylotrophus* strain CBMB147	EU194892	Bacteria	Proteobacteria	Betaproteobacteria	Methylophilales	Methylophilaceae	*Methylophilus*
*Nitrosomonas europaea* strain ATCC 19718	AL954747	Bacteria	Proteobacteria	Betaproteobacteria	Nitrosomonadales	Nitrosomonadaceae	*Nitrosomonas*
*Herminiimonas arsenicoxydans*	CU207211	Bacteria	Proteobacteria	Betaproteobacteria	Burkholderiales	Oxalobacteraceae	*Herminiimonas*
*Nitrosospira briensis* strain Nsp10	AY123800	Bacteria	Proteobacteria	Betaproteobacteria	Nitrosomonadales	Nitrosomonadaceae	*Nitrosospira*
*Cupriavidus necator* strain VKPM B-8562	JQ695937	Bacteria	Proteobacteria	Betaproteobacteria	Burkholderiales	Burkholderiaceae	*Cupriavidus*
*Thauera selenatis* strain ATCC 55363	Y17591	Bacteria	Proteobacteria	Betaproteobacteria	Rhodocyclales	Rhodocyclaceae	*Thauera*
*Hydrogenophaga flava* strain DSM 619	NR_114858	Bacteria	Proteobacteria	Betaproteobacteria	Burkholderiales	Comamonadaceae	*Hydrogenophaga*
*Sutterella wadsworthensis* strain ATCC 51579	GU585669	Bacteria	Proteobacteria	Betaproteobacteria	Burkholderiales	Sutterellaceae	*Sutterella*
*Spirillum winogradskyi* strain D-427	NR_115328	Bacteria	Proteobacteria	Betaproteobacteria	Nitrosomonadales	Spirillaceae	*Spirillum*

Organism (Non-Betaproteobacteria)							
*Dechlorospirillum* sp. strain M1	GQ262802	Bacteria	Proteobacteria	Alphaproteobacteria	Rhodospirillales	Rhodospirillaceae	*Dechlorospirillum*
*Mariprofundus ferrooxydans* strain PV-1	EF493243	Bacteria	Proteobacteria	Zetaproteobacteria	Mariprofundales	Mariprofundaceae	*Mariprofundus*
*Geobacter metallireducens* strain GS-15	L07834	Bacteria	Proteobacteria	Deltaproteobacteria	Desulfuromonadales	Geobacteraceae	*Geobacter*
*Ferrovibrio denitrificans* strain Sp-1	GQ365620	Bacteria	Proteobacteria	Alphaproteobacteria	Rhodospirillales	Rhodospirillaceae	*Ferrovibrio*
*Shewanella oneidensis* strain MR-1	NR_074798	Bacteria	Proteobacteria	Gammaproteobacteria	Alteromonadales	Shewanellaceae	*Shewanella*
*Paracoccus ferrooxidans* strain BDN-1	AY954687	Bacteria	Proteobacteria	Alphaproteobacteria	Rhodobacterales	Rhodobacteraceae	*Paracoccus*
*Thermomonas brevis* strain LMG 21746T	AJ519989	Bacteria	Proteobacteria	Gammaproteobacteria	Xanthomonadales	Xanthomonadaceae	*Thermomonas*
*Escherichia coli* strain CFT073	AE014075	Bacteria	Proteobacteria	Gammaproteobacteria	Enterobacteriales	Enterobacteriaceae	*Escherichia*
*Acinetobacter* sp. strain X9-2	KC156905	Bacteria	Proteobacteria	Gammaproteobacteria	Pseudomonadales	Moraxellaceae	*Acinetobacter*

### 2.3 PCR amplification of the 16S rRNA gene fragments

PCR amplification was conducted from environmental DNA using the newly designed primers. Each PCR reaction consisted of 0.5 μl of DNA Dream Taq polymerase (5 U/μL; Fermentas, Thermo Fisher Scientific, Waltham, MA, USA), 5.0 μL 10x Dream Taq buffer, 5.0 μL dNTPs (final concentration 0.2 mM), 5.0 μL MgCl_2_ (final concentration 2.0 mM), 0.5 μL of each primer (final concentration 5 μM), 0.5 μL (~20 ng) DNA template, 0.5 μL Bovine Serum Albumin (1 mg/mL) and nuclease free water to make a final volume of 50 μL [16]. The PCR conditions for the *Betaproteobacteria*-specific primers were as follows: initial denaturation at 95 °C for 3 min, 35 cycles of 95 °C for 1 min, 46 °C for 45 s, 72 °C for 2 min, and final extension at 72 °C for 10 min. The optimum annealing temperature was selected based on a gradient PCR approach.

All PCR reactions were performed in triplicate and subsequently pooled together. The pooled PCR products were then purified using a gel purification kit (Qiagen, Hilden, Germany) as per manufacturer's instructions.

### 2.4 Clone library construction, DNA sequencing and sequence processing

Purified PCR products were cloned using pGEM®-T Easy Vector system (Promega, Madison, WI, USA) following the manufacturer's instructions. Plasmid DNA containing the inserts was sequenced in both directions using SP6 and T7 primers in an ABI Prism 3730 Genetic Analyzer based on BigDye Terminator chemistry. Sequence chromatograms were checked manually for miss-spaced peaks, double peaks and peak shifts using BioEdit version 7.1.3 [35]. The sequences were further checked for Chimera using Bellerophon [36] and chimeric sequences were excluded from downstream analyses. Following quality control, the generated sequences were clustered into operational taxonomic units (OTU) based on 97% sequence using Mothur [37]. An OTU table was generated and subsequently used for calculating the diversity and richness indices. Taxonomy was assigned to the representative OTU sequences using the SILVA database (version 123) within Mothur. A Fisher's exact test with Storey's FDR correction for multiple comparisons was applied using the STAMP software package version 2.1 [38] to determine differential abundance of the *Betaproteobacteria* OTUs between the two wells. Corrected p*-*values<0.05 were considered significant.

### 2.5 Phylogenetic analysis

Representative sequences of the OTUs were first automatically aligned using the web-based SINA aligner [39] and imported into the ARB-SILVA database SSU Ref NR 123 [40] using the ARB software package [41]. A maximum likelihood (PhyML) tree was calculated with almost full-length 16S rRNA gene sequences (>1300 nt) of closely related reference bacteria or environmental clones based on 1188 alignment positions by using a positional variability filter for bacteria. Using the ARB parsimony tool, the OTU sequences were subsequently added to this tree one at a time by using a 50% sequence conservation filter and positional variability filters covering the individual length of each representative OTU sequence, without changing the overall tree topology. Filled circles at the node of the branches indicate lineages with >80% bootstrap support (100 re-samplings). The scale bar represents 5% estimated sequence divergence as inferred from maximum likelihood analysis. *Geobacter metallireducens* (Accession Number - L07834) was used as outgroup but was pruned from the tree. The tree was visualized using iTOL version 3 [42] and a bubble plot showing the relative sequence abundance of the OTUs was created using the R package ggplot2 [43].

### 2.6 Availability of sequences

All clone sequences generated in this study were submitted in GenBank at NCBI under Accession Numbers KY458643 – KY458731.

## 3 Results and discussion

### 3.1 Groundwater sampling and geochemical analyses

Groundwater pH, temperature and TDS were consistent with previous observations [16] whereas an increase in the total As concentration was observed in both wells (Table 2). Total Fe concentration showed an unpredictable temporal variability in both wells, whereas concentrations of dissolved nitrate and phosphate were much higher in Well 204. It is important to note that Well 204 is located within an agricultural field where local farmers routinely employ fertilizers for paddy cultivation. Leaching of fertilizer components by rainwater in monsoon and subsequent groundwater recharge could explain such elevated nitrate and phosphate concentrations in this well compared to Well 28 which is located within a household. Dissolved oxygen (DO) concentration was also slightly higher in Well 204. Previous data on these two wells have indicated evidence of terrigenous dissolved organic matter input and more oxic nature of groundwater in Well 204, suggesting faster recharge of groundwater in this well [24].

**Table 2:** Summary of three year's geochemical data comprised of groundwater pH, temperature, dissolved oxygen (DO), total dissolved solids (TDS), total arsenic (As), total iron (Fe), nitrate and phosphate in the two BDP wells.

	Well 28			Well 204		
	Y2013*	Y2011^‡^	Y2010^‡^	Y2013*	Y2011^‡^	Y2010^‡^
pH	7.3	7.3	6.9	6.9	7.3	6.8
Temperature (°C)	24.3	22.3	24.7	25.6	23.4	24.4
DO (mg/l)	2.8	n.m.	n.m.	3.1	n.m.	n.m.
TDS (mg/l)	351	358	336	382	379	373
Total As (μg/l)	81	55	35	125	117	37
Total Fe (mg/l)	1.3	1.65	1	2.4	3.45	0.39
NO_3_^-^ (mg/l)	0.5	n.m.	n.m.	5.14	n.m.	n.m.
PO_4_^3-^ (μg/l)	50	n.m.	n.m.	260	n.m.	n.m.

*This study; ‡ previous two year's sampling data; n.m.: not measured.

### 3.2 Diversity of *Betaproteobacteria* in the BDP aquifers

The specificity of the newly designed primers was investigated by targeting genomic DNA extracted from 3 *Betaproteobacteria* strains (*Hydrogenophaga atypica* strain BDP10, *Hydrogenophaga bisanensis* strain BDP20 and *Acidovorax facilis* strain BDP24) isolated previously in our laboratory from groundwater of our study site [12]. Successful amplification of a ca. 950 bp long fragment was observed for all of the above isolates. Pairwise alignment of the above amplicon sequences to the full-length 16S rRNA gene sequences of the same isolates obtained after amplification with universal primers revealed 100% sequence identity in all cases. Contrarily, amplification was not observed when genomic DNA from *Escherichia coli* (class - *Gammaproteobacteria*) and *Bacillus subtilis* (class - *Bacilli*), representing bacteria belonging to other classes, were used as template.

**Figure 2:**
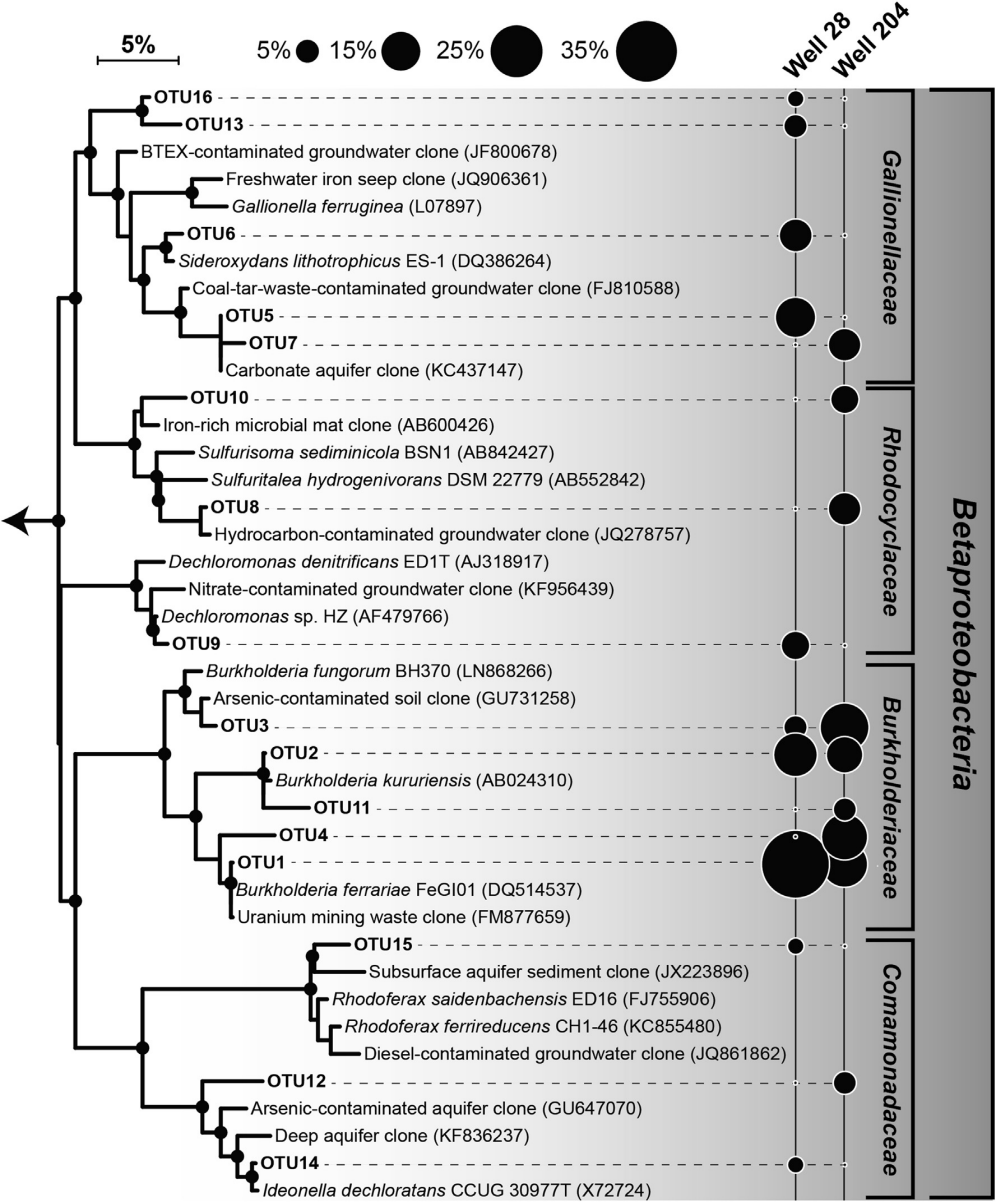
16S rRNA gene phylogenetic tree of the 16 representative *Betaproteobacteria* OTUs along with their closest phylogenetic relatives. The tree is annotated with the OTU detection frequencies in the two wells shown by the overlain bubble plot. The size of the bubbles indicates percentage relative sequence abundance. Shaded background panels show four family-level clades.

**Figure 3:**
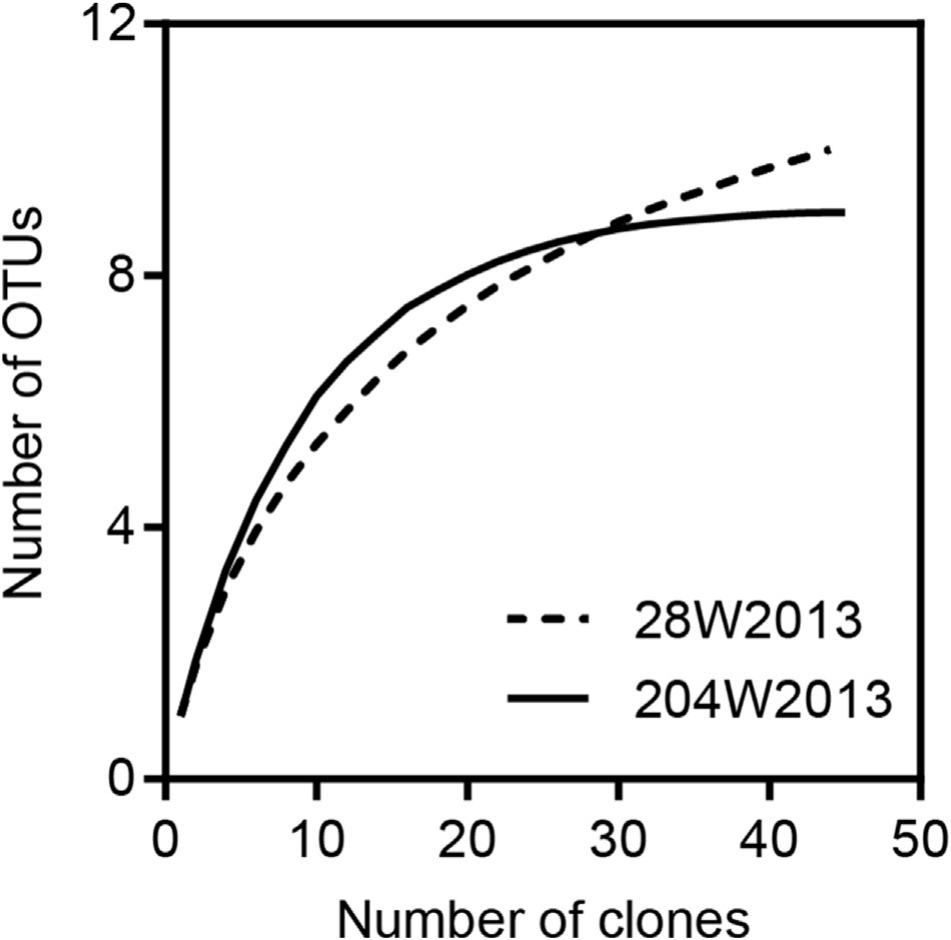
Rarefaction curves of observed *Betaproteobacteria* OTUs for the 16S rRNA gene clone libraries from the two BDP wells.

**Figure 4:**
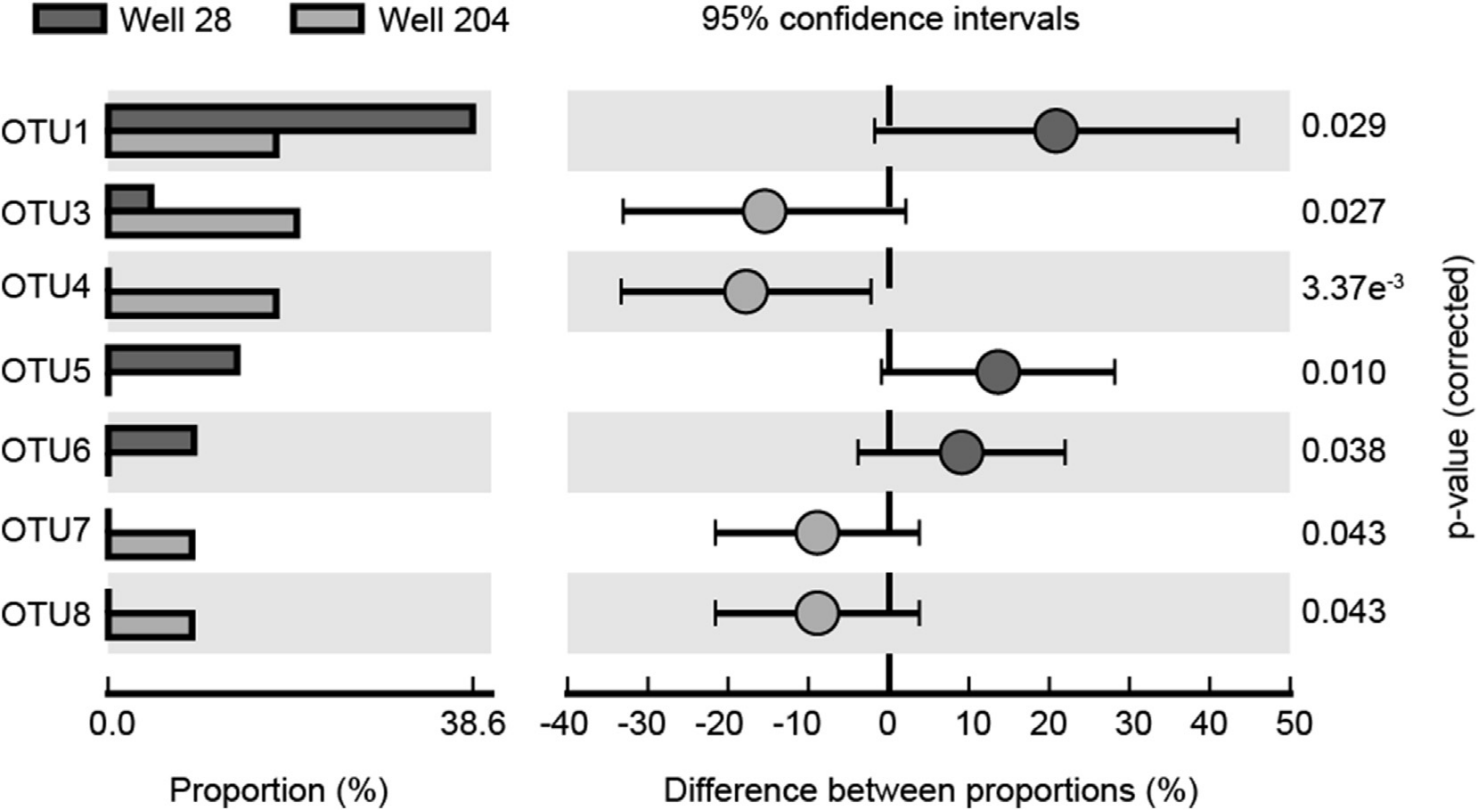
Differences in *Betaproteobacteria* communities (at the OTU level) between Well 28 and Well 204.

Two clone libraries (28W2013 and 204W2013) consisting of 44 and 45 clones respectively, were generated using the above primers in order to investigate diversity of *Betaproteobacteria* in Well 24 and Well 208. The diversity indices for both libraries have been summarized in Table 3. When all 89 clone sequences were grouped into OTUs based on a cut-off of 97% identity on nucleotide level, a total of 16 OTUs were produced (Figure 2). Taxonomic assignments placed the OTUs in four major families, *Burkholderiaceae* (5 OTUs), *Comamonadaceae* (3 OTUs), *Gallionellaceae* (5 OTUs) and *Rhodocyclaceae* (3 OTUs). *Burkholderiaceae* accounted for the majority of the clone sequences in both libraries, representing a total of 26 (59% relative abundance; 3 OTUs) and 32 (71% relative abundance; 5 OTUs) clones in libraries 28W2013 and 204W2013, respectively. Members of *Gallionellaceae* were relatively more abundant in Well 28 (29.5% relative abundance; 4 OTUs) than in Well 204 (8.9% relative abundance; 1 OTU). Conversely, clones belonging to *Rhodocyclaceae* were more prominent in Well 204 (15.6% relative abundance; 2 OTUs) than in Well 28 (6.8% relative abundance; 1 OTU). *Comamonadaceae* was the least represented family in both libraries with 4.4% (Well 28) and 4.5% (Well 204) relative abundances, respectively. The observed OTU richness was slightly higher in library 28W2013 (10 OTUs) than in 204W2013 (9 OTUs), consistent with the pattern of estimated OTU richness (Chao1 and ACE values; Table 3). Additionally, rarefaction analysis revealed a relatively greater under-saturation of the curve for library 28W2013, indicating that deeper sequencing effort is warranted to capture a greater extent of microbial diversity in Well 28 (Figure 3). Contrarily, higher Shannon index and sequence evenness values in library 204W2013 indicated greater diversity of *Betaproteobacteria* in Well 204. Our observations are consistent with data from previous years (2010 and 2011) where bacterial diversity in Well 204 was also observed to be higher [16]. Fisher's exact test comparing the two communities revealed a non-uniform pattern in OTU distribution as the abundances of 7 top OTUs (except OTU 2) were found to be significantly different across the two wells (Figure 4).

**Table 3:** Alpha diversity estimates of the *Betaproteobacteria* communities inhabiting the two BDP wells.

	Total	Well 28	Well 204
Clone sequences	89	44	45
Number of OTUs^a^	16	10	9
Chao1 richness estimate	16.75	11	9
ACE richness estimate	17.66	12.33	9
Shannon diversity index (Hʹ)	2.34	1.87	2.07
Evenness index^b^	0.84	0.81	0.94

a OTU clustering was performed based on 97% sequence identity.

b Evennness was calculated by dividing Shannon index by *Ln (OTUs)*.

The top four OTUs belonged to *Burkholderia*, a genus which was not detected in the previous investigation from these two wells when bacterial communities were analyzed using the full-length 16S rRNA and arsenite oxidase large subunit *aioA* genes as molecular markers [16]. The closest cultivated phylogenetic relative of OTUs 1 and 4 was *Burkholderia ferrariae*, which was isolated from a phosphate-containing iron ore (Figure 2) [44] and was able to generate dissolved phosphate from insoluble phosphate minerals. Such capability of mineral weathering for the purpose of nutrient acquisitioning is quite commonly observed among members of this genus. *Burkholderia fungorum*, which is a close relative of OTU 3 in our study, was indeed demonstrated to release mineral-bound arsenic in the process of solubilizing phosphate from solid-phase minerals [45]. Such ancillary mobilization of arsenic due to nutrient limitation has been hypothesized to occur conditionally depending upon the availability of poorly weathered minerals as well as supply of organic carbon. Since the BDP aquifers meet all of these criteria, it is conceivable that bacteria belonging to *Burkholderia* could play key roles in mobilization of arsenic in groundwater. It has also been demonstrated that Burkholderia can enhance arsenic toxicity by reducing methyl-arsenate compounds to toxic methyl-arsenite [46]. Interestingly, *Burkholderia* was also among the most prominent genus in recent metagenomic surveys from other arsenic-contaminated regions located in India [47] and Bangladesh [48].

*Gallionellaceae* was the next major family observed in both communities, although the overall abundance of the OTUs belonging to *Gallionellaceae* was significantly higher in Well 28 (Figure 4). Three of the 5 *Gallionellaceae* OTUs were observed to be close relatives of *Sideroxydans lithotropicus* strain LD-1, a bacterium well studied for its capability of oxidizing Fe(II) in microaerophilic conditions at neutral pH [49]. The availability of iron and low DO concentration in the BDP wells are suggestive of ideal conditions for such organisms to outcompete abiotic Fe(II) oxidation and produce biogenic hydrous ferric oxides which are known to immobilize dissolved arsenic as well as phosphate, a structural analog of arsenate. A recent molecular survey of 24 groundwater wells from Bangladesh revealed frequent occurrence of *Sideroxydans*- and *Gallionella*-like bacteria in BDP region [17]. Another study showed that the abundance of *Sideroxydans* populations increased during nitrate treatment of arsenic-rich groundwater from Cambodia [6]. Nitrate was found in both BDP wells (Table 2) and a number of genera known to contain nitrate-dependent Fe(II)-oxidizing bacteria (e.g. *Acidovorax* and *Dechloromonas*) have previously been observed in these wells. Moreover, recent cultured studies from BDP wells (204 and 28) have reported the presence of several strains of *Acidovorax*, many of which are potentially new species [12]. These results suggest that potential of *Gallionellaceae* in trapping arsenic indirectly through the production of biogenic iron oxides.

## 4 Conclusion

The findings of this study demonstrate previously undetected diversity of *Betaproteobacteria* in the BDP wells by using a novel group-specific set of primers. The relatively longer amplicon generated by these primers certainly provides greater taxonomic resolution compared to those produced by the primers that target specific variable regions of the 16S rRNA and are frequently used for generating short-read amplicon libraries using next-generation sequencing platforms. This primer set can also principally be used for designing nested PCR assays to generate *Betaproteobacteria*-specific short-read amplicon libraries by high-throughput sequencing platforms. The physiology of the dominant genera detected in this study suggests that they are potentially key players in iron oxidation and phosphate dissolution in these aquifers. Both of these microbially mediated processes immensely impact arsenic release and immobilization in the shallow aquifers of the BDP region. The results of this study suggest that in addition to their capability of oxidizing and reducing As compounds, members of *Betaproteobacteria* have the potential to impact arsenic cycling indirectly by altering the redox states and solubility of compounds that in turn affect the entrapment or release of As in the BDP aquifers.

## Declarations

### Author contribution statement

Anirban Chakraborty: Conceived and designed the experiments; Performed the experiments; Analyzed and interpreted the data; Contributed reagents, materials, analysis tools or data; Wrote the paper.

Chanchal K. DasGupta: Contributed reagents, materials, analysis tools or data; Wrote the paper.

Punyasloke Bhadury: Conceived and designed the experiments; Contributed reagents, materials, analysis tools or data; Wrote the paper.

### Funding statement

This work was supported by the Dr. D. S. Kothari Postdoctoral Fellowship awarded to Anirban Chakraborty. Punyasloke Bhadury acknowledges FIRE and ARF grants of IISER Kolkata to undertake the study.

### Competing interest statement

The authors declare no conflict of interest.

### Additional information

All clone sequences generated in this study were submitted in GenBank at NCBI under accession numbers KY458643 – KY458731.
